# Comparative Biologists and Aerospace Engineers: Important Allies to Explore the Secrets of Natural Flight

**DOI:** 10.1093/icb/icag123

**Published:** 2026-07-24

**Authors:** Jasmin C M Wong, Vikram B Baliga, Christina Harvey

**Affiliations:** School of Civil, Aerospace, and Design Engineering, University of Bristol, Bristol, BS8 1TR, UK; Department of Zoology, University of British Columbia, Vancouver, BC V6T 1Z4, Canada; Department of Mechanical and Aerospace Engineering, University of California Davis, Davis, CA 95616, USA

## Abstract

To uncover the principles behind the multifaceted topic of animal flight, we must incorporate perspectives on physics, physiology, ecology, and evolution, themes explored in the *“Dinosaurs to Drones: Comparative Approaches to Understanding Flight”* symposium. Yet these fields often remain siloed by differences in terminology and approaches. By contrasting evolution and engineering optimization, we highlight how differences between seemingly similar concepts can lead to misunderstandings, and how appreciation of those differences can be leveraged to reveal fundamental flight principles such as multifunctional constraints and adaptive versatility. These principles can then be integrated with conventional optimization methodology to both expand the design space for robust aerial systems and deepen insight into how biology achieves adaptable flight. Finally, drawing on the symposium’s panel discussion on how to successfully execute interdisciplinary endeavors, we emphasize the importance of supervisory and institutional support for establishing interdisciplinary training, collaboration, and communication opportunities.

## Introduction

Powered flight has evolved at least four times in the animal kingdom: insects, pterosaurs, birds, and bats. From each of these biological origins, hundreds to thousands of species capable of flight have diversified in physical shape and flight kinematics. Inspired by these flying animals, the Wright brothers, alongside other engineers, developed aircraft capable of powered flight over a century ago (Lilienthal 1911). Nowadays, aircraft come in many shapes and sizes, intentionally engineered for desired flight function or performance. Despite our technological achievements, certain feats of animal flight are still challenging to achieve with aircraft, such as the rapid and precise control that animals exhibit regularly (Harvey et al. 2022). For example, hawkmoth motor programs can achieve submillisecond-scale temporal precision for complex maneuvers through the coordination of relatively few muscles (Wood et al. 2024). Meanwhile, spiking neural networks designed for rapid processing and real-time response on robotic platforms remain an ongoing challenge to implement for complex tasks (Harvey et al. 2023). How, then, can we appropriately extract relevant information from the rich biological variation of flying animals to enhance future aircraft?

To fly, forces and moments in three spatial dimensions must be balanced and/or controlled. However, the differences among extant flying animals and engineered aircraft tell us that there is no single “solution” to flight, but that a variety of approaches are viable. Establishing causal relationships, or even correlations, between those solutions and flight capabilities, or “form-function relationships,” is nontrivial. As animals exemplify a proof of concept for the capabilities they demonstrate, we propose that these form-function relationships may be developed by leveraging phylogenetically informed comparisons to remove the bias caused by evolutionary links among species. In addition, the effective cross-field translations required by phylogenetically informed flight studies needs a shared framework that blends the scientific languages of aerospace engineering and comparative biology.

This work serves as an introduction to the “*Dinosaurs to Drones: Comparative Approaches to Understanding Flight*” symposium that occurred at the Society for Integrative and Comparative Biology (SICB) Annual Meeting in Portland, OR in 2026. This journal issue includes a collection of papers written by the symposium’s invited speakers. A recurring theme throughout the symposium was the potential for engineering design to leverage evolved biological variability to better understand and quantify flight. Conversely, we also discussed how analytical aerodynamic modelling (Usherwood et al. 2020), or aerial robotics (Sedky et al. 2024) through an approach called Robotics-Inspired Biology (Gravish and Lauder 2018), can serve as testbeds to question, perturb, and explore fundamental hypotheses. Our goals were to promote knowledge transfer across disparate disciplines, establish a shared language to discuss flight, and identify practical ways to translate phylogenetic insights into advances in animal flight research and aerial robotic design. These principles motivate our discussion of effective methods to train upcoming scientists and engineers to contribute at the interface of comparative biology and aerospace engineering.

In fitting with the symposium themes, this paper introduces a key confusion between aerospace engineers and comparative biologists: the differences and similarities between engineering design optimization and evolutionary processes. To illustrate our case, we will often refer back to the avian wing shape and function as it allows direct comparison to aircraft wing design.

## Evolution does not guarantee optimal form–function relationships

**Fig. 1:**
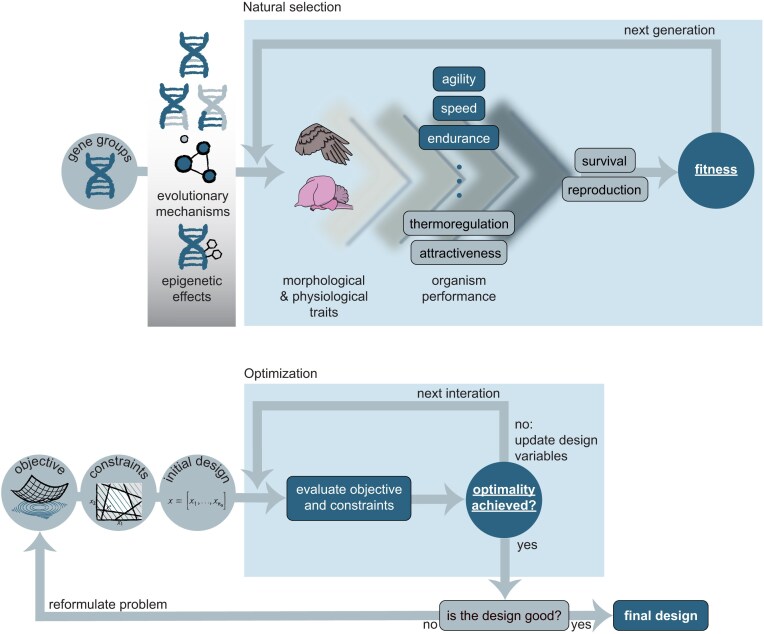
Schematics of the evolutionary process and engineering design optimization. The filled blue box area encapsulates the iterative aspects of natural selection and optimization, respectively. In evolution, there are additional evolutionary mechanisms such as mutations, recombination, and genetic drift which introduce randomized changes to the quantities (the gene groups) subject to natural selection. Relationships between traits, performance, and fitness are not explicitly defined and can variably interact with one another in a context- and time-dependent fashion. In engineering design optimization, a human designer’s input is often a part of the design cycle to ensure the optimized output achieves the designer’s goals. Relationships between variables and objective values are directly and clearly defined by analytical equations or numerical solutions.

While the term “evolution” is commonly used to describe the process driving biological variation and biodiversity, Darwin primarily describes evolution as “descent with modification” in his famed *Origin of Species*. This modification has resulted in continual diversification of flying animals over hundreds of millions of years (Glaeser et al. 2017), resulting in immense variation in size, shape, and behavior among species capable of powered flight. Evolution is an ongoing process, acting on heritable genetic variation formed through random mutations and reshuffled through genetic recombination. Through perhaps the most well-known of the evolutionary mechanisms, natural selection, the proportion of beneficial traits in a population that enhances relative fitness in a given environment is increased (Gregory 2009) (Fig. 1).

Natural selection is colloquially referred to as “survival of the fittest.” This phrase has led to a common misconception that the observed traits in extant species represent a design optimum. Flight has the potential to provide strong advantages for individual survival, and therefore, organismal fitness, by improving chances in predator-prey interactions, foraging success, finding safer habitats, and more (Alexander 2015). Therefore, it can be tempting to claim individual traits (e.g., wing area, presence of feathers) have been tuned to improve flight capabilities, even though these traits need only be sufficient for survival and reproduction to be passed on to the next generation, rather than being optimally performing. This nuance can lead to erroneous conclusions on form-function relationships, which can have cascading effects on subsequent studies on the evolution and biomechanics of flight and bio-inspired engineering.

### Historical, genetic, and developmental constraints limit the explored trait space

Evolutionary mechanisms can act only on the available heritable variation, with changes through a population accumulating over time. As a result, species that are more closely related are anticipated to have greater similarity in traits due to their relationships within an evolutionary lineage. This can be addressed through reconstructed phylogenetic trees, diagrams that quantify and visualize evolutionary relationships among species based on common features or genetic similarities (Felsenstein 2004). Nonindependence among species can then be incorporated statistically using techniques such as phylogenetic generalized least squares (PGLS; Grafen 1989), which models the expected covariance among species based on their shared evolutionary history. This method allows researchers to test for correlations between flight-relevant traits and function or performance. This is exemplified in studies on how skeletal structure, wing shape, range of motion, or feather shape relate to flight style or aerodynamic performance (Wang and Clarke 2015; Baliga et al. 2019; Walters et al. 2025; Widrig et al. 2025).

It is important to note, however, that these trees are themselves hypotheses. Phylogenetic trees are inferred statistically from genetic data, fossil evidence, and/or morphological traits. The predicted relationships are therefore biased by data availability and quality, with multiple trees being similarly possible. Furthermore, time-calibrating these phylogenies often relies on fossil or geological evidence to estimate divergence times and to place extinct taxa relative to extant lineages. Fossil data are often unevenly distributed across environments, time periods, and taxonomic groups as their preservation depends on whether conditions were conducive to fossilization; as a result, many species may have gone extinct without leaving identifiable fossil evidence. Placement of the fossilized extinct species within a clade is often based on morphology, which can be confounded by convergent evolution (Forest 2009; Sayol et al. 2020). As such, mismatches between molecular or morphology-based estimations of phylogenetic trees remains an ongoing challenge (A. Chen et al. 2025).

Further limiting the full exploration of the possible trait space to achieve globally optimized function are the environmental and competitive conditions at that time. In a letter to Joseph Hooker in 1857, Darwin writes “. . . organic beings are not perfect, only perfect enough to struggle with their competitors.” There, he is referring to a key aspect of natural selection: fitness is evaluated purely against the conditions of the time (Gould and Lewontin 1979).

### Natural selection acts on whole-organism fitness, rather than optimizing individual traits in isolation

While natural selection can produce seemingly optimized traits for particular functions, biological traits should not be assumed to represent globally optimized engineering solutions as they are shaped by evolution within historical and developmental constraints, and often subject to competing functional demands and trade-offs. As such, the resultant features are often suboptimal for a particular function. Adaptations for flight performance must be balanced against other aspects of life such as nesting behavior, parental care requirements (Dial 2003), and sexual selection (Barbosa and Møller 1999). Even different objectives of flight performance such as stability and maneuverability can impose opposing selective pressures on morphology (Harvey et al. 2022). Individual traits may be erroneously associated with limited or no functional benefit when considered in the wrong context, i.e. when evaluated outside the ecological, developmental, or functional context in which they evolved. Small proto-wings in transitional morphologies or as juveniles provide functional advantages when considered with leg-use (Heers 2016), and evidence of aerodynamic-based selection has been found to be more significant when considering only the hand-wing of birds (Rader and Hedrick 2023) or the forewing of insects (Liu et al. 2024).

Additionally, individual traits may confer limited to no advantage with respect to a particular function. They can instead arise from allometric scaling, pleiotropy, material or mechanical compensation to another functionally unrelated or indirectly related trait (Gould and Lewontin 1979). The origins of flight structures are often decoupled from the origin of flight function (Dudley and Yanoviak 2011), such as flight feathers which predate the origin of flight (Benton et al. 2019), or the acrocoracohumeral ligament which may have developed its stabilizing role only after gliding flight was achieved (Baier et al. 2007). There is, therefore, a need to analyze many “form” and “function” parameters, creating a large multidimensional data analysis problem. Techniques have been developed over the years which can facilitate interpreting high-dimensional datasets (France et al. 2025), including approaches such as PGLS that account for the effect of evolutionary relatedness on trait variation among species (Hieronymus 2015; Janisch et al. 2024), and extended methods for comparing species in a multivariate phenotype space (Adams and Collyer 2019) or evaluating functional trade-offs (Sansalone et al. 2024).

To summarize, evolutionary mechanisms do not guarantee a full exploration of the trait space nor optimality of particular traits with regard to a function, even one as important as flight locomotion. For this reason, known morphospaces do not fully cover the entire plausible theoretical morphospaces (Liu et al. 2024; Walters et al. 2025), and studies have found that some aerodynamically important wing traits do not appear to align with any particular optimum (Liu et al. 2024). As such, careful experimental design combined with explicit incorporation of evolutionary relationships is necessary to assess the relevance and degree of optimality of particular traits for flight, which can then be further investigated by engineers interested in bioinspired design.

## Engineering design optimization versus natural evolutionary processes

Natural processes, from ant colony behavior to natural selection, have inspired many algorithmic advances (Dorigo et al. 1996; Simon 2013). However, while evolution has “optimizing tendencies” (i.e., can produce outcomes that appear optimized), it is not a true optimizing routine (Taylor and Thomas 2014). Appreciating the difference between true engineering optimization and the evolutionary process is therefore necessary for effectively translating biological insights into engineered design.

To clarify these distinctions, we will describe the evolutionary process using engineering design optimization terminology as established by Martins and Ning (2021). Because optimization algorithms vary widely, we discuss a general overview of the process rather than any specific implementation. For additional details on engineering design optimization implementations and algorithms, refer to Martins and Ning (2021).

### Formulating the problem

**Fig. 2:**
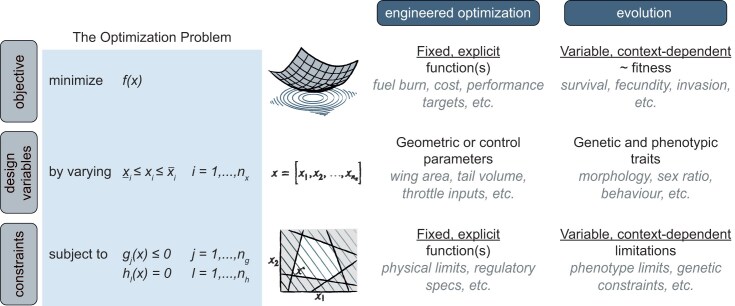
A comparison between problem formulation in engineering design optimization and a natural evolutionary process. Of note is the contrast between the fixed, and explicit aspects of engineered optimization and the variable, context-dependent nature of evolution. Graphics adapted from (Martins and Ning 2021).

In engineering optimization, we first must formulate the problem of interest by identifying the design variables, objective function, and constraints (Fig. 2).

The design variables are quantities that the optimization algorithm (hereafter: “optimizer”) can adjust at each iteration. For example, wing span and wing area may each be design variables in an aircraft wing optimization routine.

The objective function is some function of the design variables that computes and returns the variable of interest in the optimization routine. This is the value being optimized, driven either to a minimum or a maximum. It is possible to formulate the problem as a single or multiobjective problem.

Finally, constraint functions impose inequality or equality-based limits on design variables or combinations of design variables. Many algorithms allow temporary infeasibility in these constraints within each iteration, and then evaluate the constraint function value against established optimality criteria.

Certain evolutionary parameters play similar roles. Design variables may be compared to genotypes, the genetic makeup of organisms, because altering these biological instructions will often affect an organism’s morphological, physiological, or behavioral characteristics (collectively known as “phenotype”). However, unlike the established interdependent relationships between engineered design variables, the relationship between genotype and phenotype is not straightforward. Genes can influence multiple traits, be “read” or modified in different, yet heritable, ways (epigenetics) as determined by parental or social behavior and other environmental factors (Ashe et al. 2021), and can interact and influence one another. These mechanisms may result in a variety of observed characteristics (phenotypes) despite the same base genetic code (Merlin and Liedvogel 2019).

The objective function of evolution is often considered to be some fitness metric(s). Fitness is a time-dependent measure resulting from the interplay of multiple parameters, of which survival and fecundity are two major signals, that are difficult to combine into a single metric. As such, evolutionary processes are most comparable to multiobjective optimizations, where individual objectives may be differentially weighted over time; a trait that was beneficial in the Cambrian era may confer no benefit or even be detrimental today. This is particularly relevant today as anthropogenic changes in the environment influence behavioral and physiological responses linked to fitness (Madden et al. 2026).

Finally, the evolutionary process is constrained by many attributes, including ecological energy limits and genetic heritability, which can vary across lineages, environments and evolutionary timescales. In particular, this symposium highlighted that phenotypes are not guaranteed to be evenly distributed in extant species, and not all theoretically possible phenotypes may be genetically or developmentally accessible (Castle et al. 2021).

### Establishing the initial conditions

Once the problem has been formulated, the optimizer requires a value for each design variable to evaluate the initial state. In engineered design, these starting points can be algorithmically selected from a randomly generated seed in the design space, or informed by previous successful solutions or the designer’s best educated guess. Often a designer will try multiple initial values to ensure that the solution is not sensitive to this selection or to determine whether the solution is stuck in a local optimum (Martins and Ning 2021).

The evolutionary process is also conditioned by “initial conditions,” existing genetic and epigenetic states. This could be an early member of the avian lineage such as a Jurassic avialan like *Archaeopteryx*. Because these early avialans evolved within the tetrapod lineage, they and all modern birds inherited a conserved genomic and developmental architecture associated with the tetrapod body plan, with two forelimbs and two hindlimbs. Evolutionary innovation in avian flight occurred largely through regulatory and developmental modifications resulting in variation in genes or in developmentally and environmentally driven gene expression among populations of *Archaeopteryx*, providing some of the initial values for the evolutionary process of modern birds.

### Evaluating the objective function and constraints

Next, in engineered design, the initial values get passed into an analysis block, which computes the current value of the objective and constraint functions. For an aerodynamic-based optimization, the analysis block could use initial values of wing span and area to compute cruise aerodynamic efficiency or fuel burn. The value of the constraints, such as the aspect ratio (e.g., AR < 10) would be computed as well.

In evolutionary processes, a comparable analysis block would evaluate the outcome of each generation in a population. Unlike engineering design optimization, this evaluation does not calculate and return a quantifiable continuous value of an “objective”; organisms may die prior to procreating, eliminating genetic material from subsequent generations, or they may successfully live to reproduce and contribute to the gene pool. Survival to reproduction may be a strong filter for passing morphological or physiological information forward to next generations, whereas engineering optimization can reliably pass on information about design variables that fail or perform poorly to inform subsequent iterations. However, evolutionary outcomes are not strictly determined by the binary distinction between survival and death. This is because trait prevalence is often considered at a population level through variation in reproductive success, offspring quality, and the timing and context of reproductive events across all individuals. All of these processes contribute to a graded difference in population fitness (Gregory 2009).

### Checking for optimality

Following this analysis block, the computed objective and constraint values are compared to the optimizer’s optimality conditions. In engineering optimization, candidate solutions are explicitly evaluated against predefined optimality conditions, and convergence is determined by formal stopping criteria or fixed computing limits. For gradient-based algorithms, optimality has strict mathematical definitions such as stationarity, complementarity and feasibility, whereas some gradient-free methods use convergence metrics to weakly define stationarity. Most methods quantify how big of a change there is between the current state and previous iterations, and once that change is below a set threshold, the optimizer will be considered to have converged to an optimum. When the optimality conditions are satisfied within the prescribed tolerance, the loop stops and the calculated state is returned as the optimal solution. Note that this optimal solution is not necessarily the global optimum, and converging to a local minima is a common challenge in engineering.

Evolutionary processes, in contrast, do not inherently have an explicit stopping condition or a fixed objective function against which progress is evaluated. Evolutionary dynamics, however, can be mathematically modeled as movement on fitness landscapes, with relevant fitness functions that are typically implicit, context-dependent, and shaped by ecological and frequency-dependent interactions (Otto and Day 2011).

While evolutionary models may exhibit equilibria, such as fixation, polymorphism, or evolutionarily stable strategies (ESS), these states are not “checked” or selected by the process itself (Smith and Price 1973). Selective pressures change as populations and environments co-evolve, producing behavior that can resemble a stable yet continually adapting system (Thompson 2019). As a result, evolutionary outcomes are strongly contingent on historical and initial conditions, rather than converging reliably on a single global optimum. Instead, populations may approach, depart from, or fluctuate around such ephemeral states as selective pressures and environmental conditions change.

Consequently, evolutionary change reflects ongoing competition in a shifting landscape rather than convergence to a well-defined, fixed optimal solution. Although selection can produce locally adaptive outcomes, neither global nor permanent optimality is guaranteed, and evolutionary trajectories remain contingent on historical, ecological, and genetic conditions. This path-dependent and context-sensitive dynamic stands in contrast to engineering optimization, where the objective function and feasibility constraints are specified in advance.

### Updating the design variables

While the optimality conditions remain unmet, the engineered optimizer selects a new set of design variables and the cycle continues. Different optimization algorithms vary how the optimizer selects the next iteration’s design variables, combining knowledge of the current design variables, objective function, and constraints, using some combination of a local or global search of the space, heuristics or mathematical evaluations, and allowing for stochasticity to minimize the risk of falling into local minima. One example is a method called “elitism,” used by many genetic algorithms, where only the most fit members of a population of considered designs are retained.

This step of the optimization process is perhaps where engineered algorithms have been most directly inspired by biological processes. Even then, the evolutionary dynamics of traits at a population level are additionally shaped by complex forces that may be difficult or unbeneficial to implement in an engineered algorithm. For instance, these forces include genetic drift and gene flow, evolutionary mechanisms that change the frequency of gene variants over time within a population which arise from finite population sizes and population structure rather than from any directed search mechanism. Another difference is that evolutionary transformations may be constrained by the consequences of phenotypic transitions on fitness, either throughout an individual’s lifetime or more gradually through generational changes. Consider that small proto-wings and downy feathers may not have permitted the flight control we observe in adult, extant birds. If these features had been disadvantageous, the evolutionary transformations may have halted before reaching a stage where powered flight could be achieved. Instead, ontogenetic studies suggest that these intermediate features may have provided other functional advantages which allowed for their continued development. Meanwhile, engineered algorithms can allow for large jumps in design variables between iterations which can prevent the optimizer from getting stuck in local minima or blocked by intermediate, infeasible solutions (Heers and Dial 2012).

### Engineering calculates, evolution adapts

To summarize, there are many similar and different features between engineering design optimization and evolution. While this text has represented engineering approaches as deterministic and quantifiable, it is worth noting that many real-world problems involve complex physical interactions that are challenging to represent with functions and are often assessed empirically. As such, modern designs still struggle to entirely remove a human designer from the optimization loop (Fig. 1). Evolutionary processes, on the other hand, notably lack fixed, explicitly quantifiable objectives and constraints, statically enforced optimality criterion, or evidence of a designer providing direction.

## Symposium outcomes

### Translating evolutionary insights into engineered systems

Despite the differences between optimization methods and evolution, the connections between the two provide a powerful tool for answering novel research questions and probing hypotheses to identify underlying form-function principles within a population. As Maynard Smith aptly summarized, *“Optimization models [. . .] serve to improve our understanding about adaptations, rather than to demonstrate that natural selection produces optimal solutions”* (Smith 1978). It is only once we can identify and understand these biological adaptations that we have advanced our ability to extract a fundamental principle or mechanism for bioinspired applications.

While one approach to bioinspired design involves sifting through the variation in nature to find a species that seems particularly good at a task, the alternative approach of using a comparative or evolutionary method has become more prevalent (Ng et al. 2021). Although it is still not widely used, these comparative methods have led to major technological developments including gecko foot-inspired adhesion (Garner et al. 2019; Higham et al. 2019), butterfly scales-induced wing cooling (Tsai et al. 2020), and moth wing-inspired sound absorption (Neil et al. 2020). It is important to note that even these success stories only make use of a small subset of species, often restricted to a single model genus, indicating that further insight could be acquired with expanded comparative studies (Ng et al. 2021).

We propose that, given the diversity in flight-capable species spanning across multiple lineages from different origins of powered flight, phylogenetically and ecologically informed comparative methods (Garland et al. 2005) would provide a good starting design space for further optimization of low-Reynolds flight designs (D. Chen et al. 2025).

### Training mentees at the interface

Discussions of this manner with both biologists and engineers often require a high amount of context switching and a familiarity with both disciplines, which can feel exclusionary to newcomers. As part of our symposium’s final panel, we posed a question to panelists about how students can be trained to work at the interface of these fields. This is particularly important as despite the popularity of “bioinspired design” in research, there is often very little integration between the two disciplines. A study of the Web of Science database using rather lax selection criteria showed that only 41% of bioinspired and biomimetics research featured an author from a biology-related department (Ng et al. 2021), neglecting the surfeit of knowledge biology research produces.

In the panel discussion, commonly mentioned ways to support trainees included providing an inclusive environment for discussion, promoting diversity of thought within a research group, and developing opportunities to network between departments. Commonly mentioned barriers to interdisciplinary collaboration were difficulties adjusting to jargon and differing levels of methodological rigor, objectives, and expected outputs between fields, all of which make communication and goal alignment difficult (Newman 2024). For example, engineers may be predisposed to application-based objectives and are more used to publishing incremental results in conference papers. Exasperating this issue is that some researchers may be hesitant in going beyond their comfort zone at the risk of being seen as “less competent” with the perception that interdisciplinary researchers are a “jack of all trades, master of none” (Newman 2024). We suggest that this narrative should change to instead celebrate these researchers’ developed specialties and expertise in interdisciplinary work, communication, and collaboration. At a supervisory level, we encourage highlighting that interdisciplinary research is, in itself, a specialty and normalizing a “no bad questions” attitude. It has also been suggested that active recruitment of team members from different backgrounds with different perspectives (who are open to learning new perspectives as well) indirectly contributes to creating this open-minded environment (Moirano et al. 2020). Several panelists pointed out that while mentees should be open to cross-discipline training, supervisors must remain flexible and open themselves to the goals of individual mentees and adjust expectations accordingly.

On an institutional level, our panel highlighted the need for interdisciplinary and interdepartmental education, networking, and overall interaction. Project-based education and problem solving in teams would be useful to train students to integrate different perspectives and look at a broad range of solutions (Moirano et al. 2020). There can be a perception that interdisciplinary work involves higher mental or material overheads due to needing to learn a new field. Collaborations are critical for reducing these overheads and can be supported through internal workshops and grants (Newman 2024). University-based training programs provide an early opportunity for students to work in multidisciplinary teams at the interface of nature and engineering. Successful examples of these include an MSc program at the University of Bristol that required co-supervision from both environmental monitoring and aerial robotics academics and a National Science Foundation-supported Integrative Graduate Education and Research Traineeship at UC Berkeley which provided interdisciplinary learning and facilities for collaborative projects. On a larger scale, Horizon Europe (Patterson et al. 2023) and the Human Science Frontiers Project have been key supporters for forming cross-disciplinary research teams. As we continue to tackle challenging and novel problems, the number of truly interdisciplinary research teams must increase, requiring more institutional and federal support to become available for the continuation and proliferation of these interdisciplinary opportunities.

## Conclusion

To conclude, natural evolutionary processes may follow some optimization-like principles as engineering methods but the adaptations observed do not guarantee a “solution” that is either globally or locally optimal. This is where engineering methods can be used to further explore the design space and identify solutions that are relevant within engineering contexts but informed by biological adaptations. For this approach to be successful, the scientific community must foster interdisciplinary training, stronger communication, and an inclusive environment both at the supervisor-mentee level and on a larger institutional or community level.

## Data Availability

No new data were generated or analysed in support of this research.

## Notes

From the symposium : “Dinosaurs to Drones: Comparative Approaches to Understanding Flight" presented at the annual meeting of the Society for Integrative and Comparative Biology, January 3-7th , 2026.
